# Mild Myopic Astigmatism Corrected by Accidental Flap Complication: A Case Report

**DOI:** 10.4103/0974-9233.58414

**Published:** 2009

**Authors:** Daoud C Fahed, Charbel D Fahed

**Affiliations:** Ophthalmic Consultants of Beirut, ForeSight Foundation, Lebanon

**Keywords:** Accidental Flap Complication, Laser In-situ Keratomileusis, Myopic Astigmatism

## Abstract

A 35-year-old female presented for laser in-situ keratomileusis (LASIK). Her preoperative eye exam was normal, with a preop refraction of OD −2.50 D Sph +1.25 D Cyl ×175 and OS −2.75 D Sph +1.50 D Cyl ×165 (cycloplegic and manifest), with 20/20 BCVA OU. The central pachymetry reading was 553 μm in the right eye. Preoperative topography was normal. At the start of the pendular microkeratome path, some resistance was felt, but the microkeratome continued along its path. Upon inspection of the flap, there was a central rectangle of intact epithelium with two mirror-image flaps on both sides. The flap was repositioned and LASIK was discontinued. The cornea healed with two faint thin linear vertical parallel scars at the edge of the pupil. Postoperative inspection of the blade revealed central blunting. One month postoperatively, the uncorrected visual acuity (UCVA) was 20/20. Manifest and cycloplegic refractions were plano. This is an interesting case of accidental flap complication resulting in the correction of mild myopic astigmatism.

## INTRODUCTION

Laser *in-situ* keratomileusis (LASIK) is an effective technique for the treatment of refractive errors. Today, it is still the most commonly performed refractive procedure,^1^ despite the recent shift towards surface ablation. Flap complications secondary to microkeratome use remain a feared complication. These complications are fortunately much less frequent with the newer, third generation microkeratomes and the development of the femtosecond laser.^2^

Flap creation is known to create aberrations that can change the visual acuity; these aberrations are even more when the flap is irregular.^3^,^4^ Though, parallel T-cuts are known to correct astigmatism; to the best of our knowledge, there are no reports of it correcting myopic astigmatism^5^–^7^ other than a case report of change in refraction after traumatic injury to the cornea.^8^

We report a case where the error of refraction that was intended to be corrected by LASIK was corrected by the accidental flap complication, without the use of the excimer laser.

## CASE REPORT

**Figure 1:**
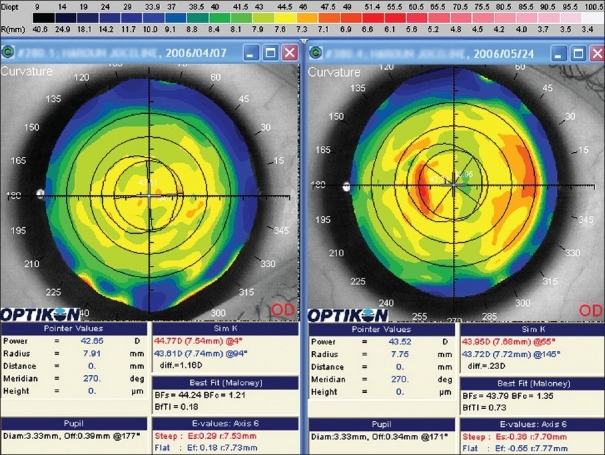
Preoperative and postoperative corneal topography

A 35-year-old female presented for LASIK to both eyes. Her past medical history was negative. Her preoperative refraction was OD −2.50 DSph +1.25 D Cyl ×175 and OS −2.75 DSph +1.50 D Cyl ×165 (cycloplegic and manifest). She had a 20/20 best corrected visual acuity (BCVA) in both eyes. The central pachymetry was 553 and 555 μm in the right and left eyes, respectively. The anterior segment exam and the dilated fundus exam were non-revealing. The Schirmer's test was >30 mm/5′ in both eyes. Preoperative topography was normal [Figure 1]. The keratometric readings were 44.77/43.51 × 94 in the right eye and 44.92/43.62 × 76 in the left eye.

LASIK was planned for both eyes using the Carriazo pendular microkeratome (Schwind, Kleinostheim, Germany) with the 9 mm ring and a superior hinge.

During the microkeratome path of the right eye, and after reaching adequate suction, the surgeon noticed that the cutting of the keratome was halted at the beginning of its path following which he took off his foot from the pedal and pressed the pedal again. The microkeratome continued its path forward and backward despite the initial resistance. The flap was found to have a central rectangle of intact epithelium with two mirror-image flaps on both sides. LASIK was discontinued, the torn flap was repositioned in place and a bandage contact lens applied on the corneal surface. The patient was informed of the flap complication and about the need to postpone the LASIK. She was treated with a combination of 0.1% dexamethasone (Dexa-Sine, Alcon-Couvreur) and 0.3% ciprofloxacin (Ciloxan, Alcon Inc) QID, in addition to artificial tears hourly.

**Figure 2:**
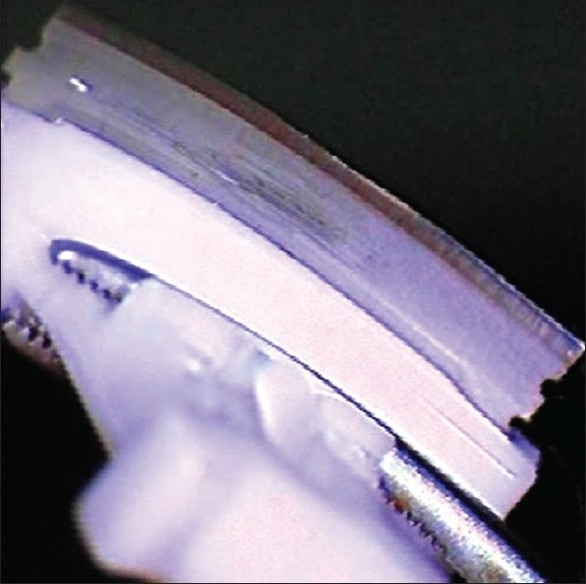
Microkeratome blade (notice the blunting in the center)

Inspection of the blade revealed central notching and blunting due to its rubbing on the guard flange of the ring [Figure 2]. The protective flange of the ring was found to be bent inward, probably from trauma inflicted by the technician to the ring while cleaning it.

**Figure 3:**
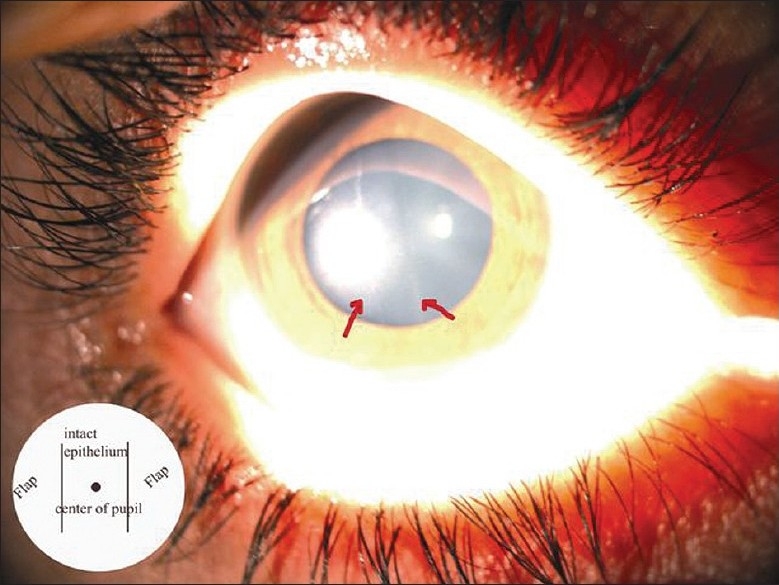
Postoperative corneal photograph. Inset: Diagrammatic representation of flap

Two days later, two thin, faint, linear vertical parallel scars at the edge of the pupil were seen [Figure 3] and the patient had an UCVA of 20/40. Two weeks postoperative, the UCVA was 20/20. Manifest and cycloplegic refractions were plano. The vision remained stable at the 3 and 6 months postoperative visit. The patient then underwent an uncomplicated LASIK procedure in the left eye with good result at the 3 months postoperative visit.

## DISCUSSION

This case illustrates the need to approach each step of the LASIK procedure carefully. The surgeon should have stopped the procedure when he felt the resistance, but fortunately the end result had no detrimental effect on the patient. We believe that following events results in this complication. The shaft of the pendular microkeratome ring was bent by trauma. The blade of the Carriazo Pendular Keratome rubbed on the metal flange at its center, resulting in a notching and blunting of its cutting edge centrally [Figure 2]. This blunting prevented proper cutting, and the result was a flap of residual corneal tissue in the central cornea. The parallel corneal cuts acted like two central parallel T-cuts, which resulted in the correction of the patient's astigmatism and myopia.

Radial keratotomy (RK) and astigmatic keratotomy have been widely used in the past to correct ametropia. The Prospective Evaluation of Radial Keratotomy Study (PERK study) evaluated the efficiency and outcome of RK.^9^ However, the low predictability of results and the high diurnal fluctuation of vision resulted in limited use of these incisional surgeries, and they were replaced by the excimer laser.^10^ Khokar *et al.* reported the correction of astigmatism by opposite, clear corneal incisions. 7 A recent case report describes the self resolution of myopia secondary to ocular trauma.^8^ Incisions in the cornea increase the higher order aberrations. We previously reported on the increase in higher order aberration post-flap creation and the influence this will have on visual outcome.^11^ The vision correction in this patient was probably due to a change of refraction secondary to the T-cuts, and not from the higher order aberrations that were not markedly altered (data not included). Despite the fact that the cuts in the ‘T-cuts procedure’ are deeper, our results show an alteration in the refraction after the procedure. This change in refraction occurred because of changes in collagen fibrils. The mid-peripheral corneal lamellae extending from limbus to limbus were cut, and this resulted in alteration of the corneal curvature [Table 1].^12^ The incisions created in the superficial stroma flattened the horizontal curvature and steepened the vertical curvature thus antagonizing the myopic astigmatism in the right eye of the patient. This case is an example of a flap complication resulting in desirable outcome for the patient.
